# Impact of Osteocalcin on Glycemic Regulation and Insulin Sensitivity in Type 2 Diabetes Mellitus Patients

**DOI:** 10.7759/cureus.71675

**Published:** 2024-10-17

**Authors:** Ritu Tiwari, Shraddha Singh, Manish Bajpai, Narsingh Verma, Shivam Verma

**Affiliations:** 1 Physiology, King George's Medical University, Lucknow, IND

**Keywords:** biomarkers, glycemic control, homa-ir, insulin resistance, osteocalcin, type 2 diabetes mellitus

## Abstract

Background

Type 2 diabetes mellitus (T2DM) is a worldwide health issue impacting millions of individuals. In recent years, bone has been identified as an endocrine organ that regulates glucose metabolism by the release of osteocalcin, an osteoblast-specific hormone, which affects fat accumulation and blood glucose levels. Osteocalcin has been associated with insulin sensitivity and glucose control.

Objective

The study investigates the relationship between circulating osteocalcin levels with glycemic control parameters and insulin resistance in T2DM patients.

Methods

A total of 234 subjects were recruited, including T2DM patients (n=117) and age-sex-matched controls (n=117). Fasting blood samples were collected to measure fasting blood sugar (FBS), insulin, glycated hemoglobin (HbA1c), and osteocalcin levels. Osteocalcin levels were determined using an enzyme-linked immunosorbent assay. Insulin resistance was calculated using the Homeostatic Model Assessment for Insulin Resistance (HOMA-IR).

Results

The levels of osteocalcin in T2DM patients were significantly lower (7.07 ± 3.80 ng/mL) than in healthy controls (20.41 ± 13.50 ng/mL, p<0.0001). A significant negative correlation was observed between osteocalcin and HbA1c (r=-0.710, p<0.01), as well as between osteocalcin and FBS (r=-0.676, p<0.01). T2DM patients also showed significantly higher insulin resistance, as evidenced by their elevated HOMA-IR scores (4.39 ± 1.95 vs. 3.62 ± 1.82, p=0.002). There was a negative correlation between osteocalcin and HOMA-IR (r=-0.324, p=0.0001).

Conclusion

This study shows that osteocalcin levels are significantly reduced in patients with T2DM and demonstrate a negative correlation with HbA1c, FBS, and insulin resistance.

## Introduction

Type 2 diabetes mellitus (T2DM) has become a leading global health challenge, currently affecting around 425 million individuals worldwide, with approximately 90% of these cases being T2DM [1]. As a chronic metabolic disorder, T2DM is characterized by insulin resistance and hyperglycemia, resulting in various long-term complications such as cardiovascular diseases (e.g., heart attacks and strokes), diabetic retinopathy, nephropathy, and peripheral neuropathy [2]. The management of T2DM has traditionally focused on key metabolic organs, including the pancreas, liver, skeletal muscle, adipose tissue, and kidneys [3]. These organs are targeted to enhance glucose homeostasis and improve insulin sensitivity in patients.

The skeleton, long known for its structural and protective roles, is now recognized as an endocrine organ that actively participates in metabolic regulation [4]. This paradigm shift has opened new avenues for research into the role of bone-derived hormones, known as osteokines, in regulating energy metabolism [5]. Among these osteokines, osteocalcin, a hormone secreted by osteoblasts, has garnered particular interest for its influence on glucose and fat metabolism [6]. Osteocalcin promotes insulin secretion by pancreatic β-cells and enhances insulin sensitivity in peripheral tissues such as skeletal muscle and adipose tissue, thereby playing a critical role in maintaining glucose homeostasis [7]. Despite these promising findings, the molecular mechanisms underlying osteocalcin's effects on glucose metabolism remain poorly understood, particularly in human populations.

This osteocalcin-adiponectin axis may represent a novel mechanism by which the skeleton influences systemic metabolic health, highlighting the interconnected nature of bone and adipose tissue in regulating energy homeostasis [8]. Despite the growing body of evidence linking osteocalcin to glucose metabolism, significant gaps remain in our understanding of its role in T2DM. Animal models have provided valuable insights into these relationships [9,10]. Human studies are essential to confirm the translational potential of these findings. The present study aimed to investigate the correlations between osteocalcin, fasting blood sugar (FBS), glycated hemoglobin (HbA1c), insulin, and insulin resistance in T2DM.

## Materials and methods

Selection of subjects

This case-control study involved a total sample size of 234 individuals, categorized into diabetic patients (n=117) as the case group and healthy individuals as the control group (n=117). The study was conducted following the diagnosis of T2DM as the American Diabetes Association (ADA) guidelines [11].

Inclusion criteria

The inclusion criteria for the case group consisted of diabetic patients diagnosed with T2DM between the ages of 20 and 70, with diagnoses based on WHO criteria [12], including FPG and HbA1c values. The control group included healthy individuals matched with the case group based on age and sex, with no history of chronic ailments such as diabetes, autoimmune disorders, or other chronic illnesses. Control subjects were also screened to ensure they were free from any known metabolic diseases.

Exclusion criteria

Patients with type 1 diabetes mellitus and individuals with anatomical deformities such as severe scoliosis or limb deformities may be excluded from certain medical studies. This is due to the potential impact these conditions can have on respiratory function and exercise capacity measurements. Similarly, severe respiratory system deformities, including major chest wall deformities, could also lead to exclusion because they may interfere with the accurate assessment of respiratory health and physical endurance. Pregnant women were also excluded from the study. Additionally, individuals suffering from chronic illnesses such as thyroid disorders, renal disease, autoimmune disorders, or cancer were also excluded. Patients undergoing glucocorticoid therapy were excluded due to the potential impact of these medications on metabolic processes.

Diagnostic criteria for T2DM

The diagnostic criteria used in the study followed the ADA guidelines [13]. The diagnostic parameters included an HbA1c value of ≥6.5%, which reflects average blood glucose levels over the previous two to three months and is considered diagnostic for diabetes. FPG was measured after an overnight fast of at least eight hours, with a level of ≥126 mg/dL confirmed on two separate occasions indicating a diagnosis of T2DM. Additionally, patients with diabetes insipidus were excluded from this analysis to avoid confounding factors, as the pathophysiology and management of diabetes insipidus differ significantly from those of diabetes mellitus and because these patients also have polydipsia and polyuria.

Ethical considerations

The study protocol was reviewed and approved by the Ethical Committee of King George's Medical University, Lucknow (approval number: 125th ECMIIB-Ph.D/P1). Written informed consent was obtained from all participants before their enrollment in the study. The participants were informed about the study objectives and procedures, and they were free to withdraw at any point without any repercussions. A structured proforma collected relevant medical, family, and personal histories for all subjects.

Biochemical assessments

Fasting blood samples were collected from the diabetic and control groups after an overnight fast of 10-12 hours. The biochemical assessments conducted included fasting glucose and insulin levels measured using the same automated biochemistry analyzer (Cobas 6000, Roche Diagnostics, Indianapolis, IN, USA). These values were used to calculate insulin resistance using the Homeostatic Model Assessment for Insulin Resistance (HOMA-IR). HbA1c levels were measured using the BIO-RAD Variant II Hemoglobin Testing System.

Insulin resistance (HOMA-IR) calculation

Insulin resistance was calculated using the following formula:

HOMA-IR = [fasting insulin U/L) [fasting glucose mmol/L]/ {22.5}

This method estimates insulin resistance by combining fasting insulin and glucose measurements. Higher HOMA-IR values are indicative of increased insulin resistance, which is a hallmark feature of T2DM [14].

Measurement of circulating osteocalcin levels

**Figure 1:**
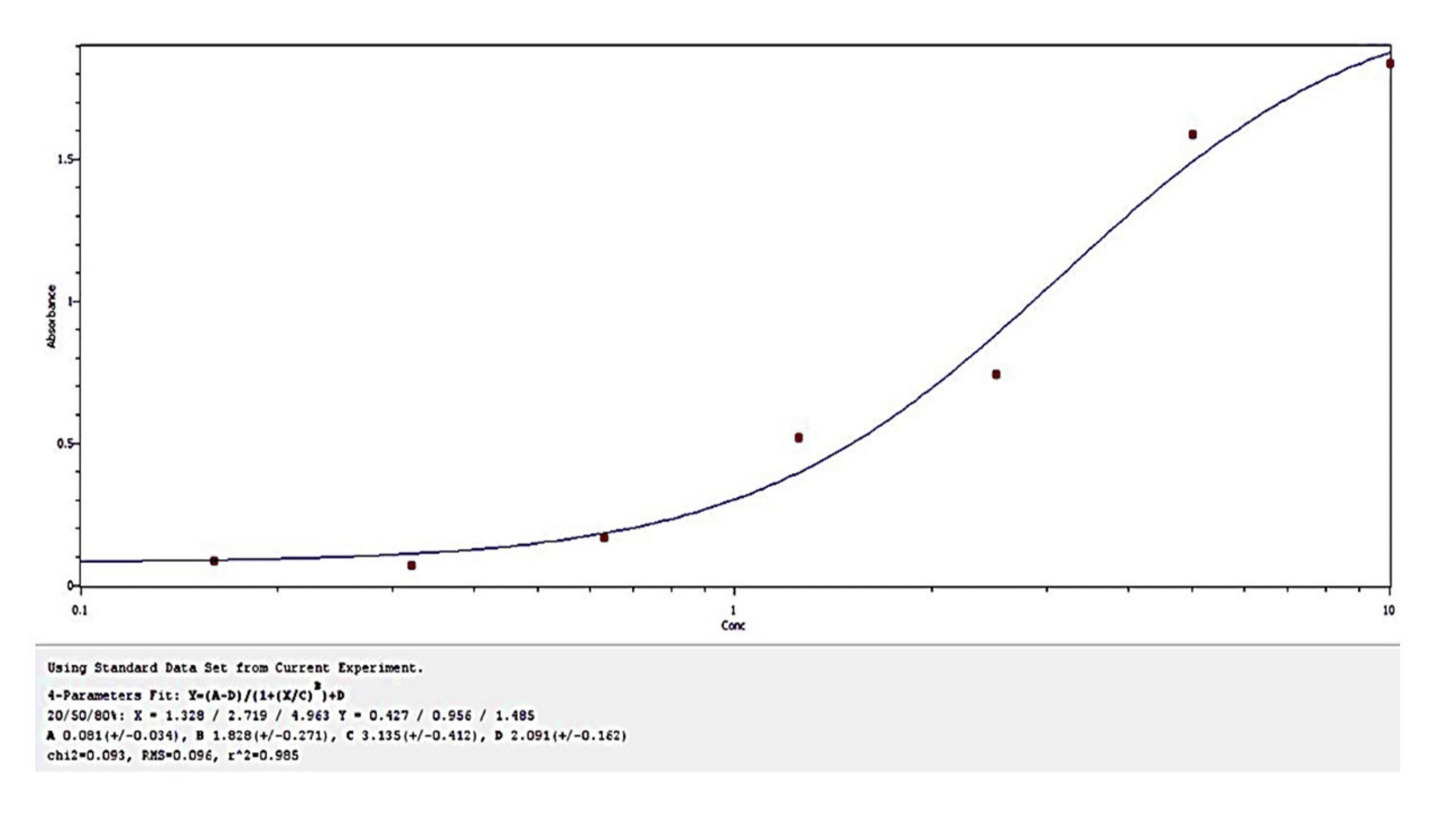
Standard curve for human osteocalcin ELISA ELISA: enzyme-linked immunosorbent assay

Circulating osteocalcin levels were measured using an Elabscience Human ucOC (Undercarboxylated Osteocalcin; Catalog No. E-EL-H611) enzyme-linked immunosorbent assay (ELISA) Kit, following the manufacturer’s instructions. All reagents were brought to room temperature (18-25°C) before use. The normal detectable range for osteocalcin was 6.25-400 pg/µL (Figure 1).

Statistical analysis

Demographic data was reported in numerical and percentage formats. The mean ± SD was employed for continuous variables, and the t-test was utilized to get the p-value. The Pearson correlation coefficient was employed to assess the relationship between the variables. A p-value <0.05 was considered statistically significant. All data were analyzed utilizing SPSS Statistics version 21.0 (IBM Corp. Released 2012. IBM SPSS Statistics for Windows, Version 21.0. Armonk, NY: IBM Corp.).

## Results

General characteristics of the study population

The mean age of diabetic participants was 42.08 ± 17.88 years, while that of the control group was 39.74 ± 17.30 years, with no significant difference between the groups (p=0.31). Gender distribution was similar between groups, with males making up 66 (56.4%) of the diabetic group and 62 (53.0%) of the control group (p=0.69). A significant difference was observed between the two groups regarding socioeconomic status (p=0.014). Among diabetic participants, 31 (26.5%) were classified as poor, 58 (49.6%) as middle class, and 28 (23.9%) as rich, compared to 36 (30.8%) poor, 37 (31.6%) middle class, and 47 (37.6%) rich in the control group (Table 1).

**Table 1:** General characteristics of the study population The chi-test was used to calculate the p-value. * p-value <0.05 was considered as statistically significant.

Variables	Diabetic (n=117) n (%)	Control (n=117) n (%)	p-value
Age (years) (mean ± SD)	42.08 ± 17.88	39.74 ± 17.30	0.310
Range (years)	38-65	25-51	
Gender			0.694
Male	66 (56.4)	62 (53.0)	
Female	51 (43.6)	55 (47.0)	
Socioeconomic status			0.014*
Poor	31 (26.5)	36 (30.8)	
Middle	58 (49.6)	37 (31.6)	
Rich	28 (23.9)	44 (37.6)	

Status of glycemic markers and osteocalcin levels in diabetic patients

There were significant differences in the glycemic markers between the diabetic and control groups. Diabetic participants had higher FBS levels (125.21 ± 14.64) compared to controls (93.64 ± 7.23 mg/dL, p<0.0001). Insulin levels were also significantly elevated in diabetic patients (15.41 ± 7.43) than in the control group (13.10 ± 3.42 IU, p=0.002). Insulin resistance was higher in diabetic patients (4.39 ± 1.95) versus controls (3.62 ± 1.82), also showing a significant difference (p=0.002) (Table 2).

**Table 2:** Glycemic parameters and osteocalcin levels of the study population The student t-test was used to calculate the p-value. * p-value <0.05 was considered as statistically significant. HbA1c: glycated hemoglobin, FBS: fasting blood sugar, HOMA-IR: Homeostatic Model Assessment for Insulin Resistance

Variables	Diabetic (n=117) mean ± SD	Control (n=117) mean ± SD	p-value
FBS (mg/dL)	125.21 ± 14.64	93.64 ± 7.23	<0.0001*
Insulin (IU)	15.41 ± 7.43	13.10 ± 3.42	0.002*
HOMA-IR	4.39 ± 1.95	3.62 ± 1.82	0.002*
Osteocalcin (ng/mL)	7.07 ± 3.80	20.41 ± 13.50	<0.0001*

**Figure 2:**
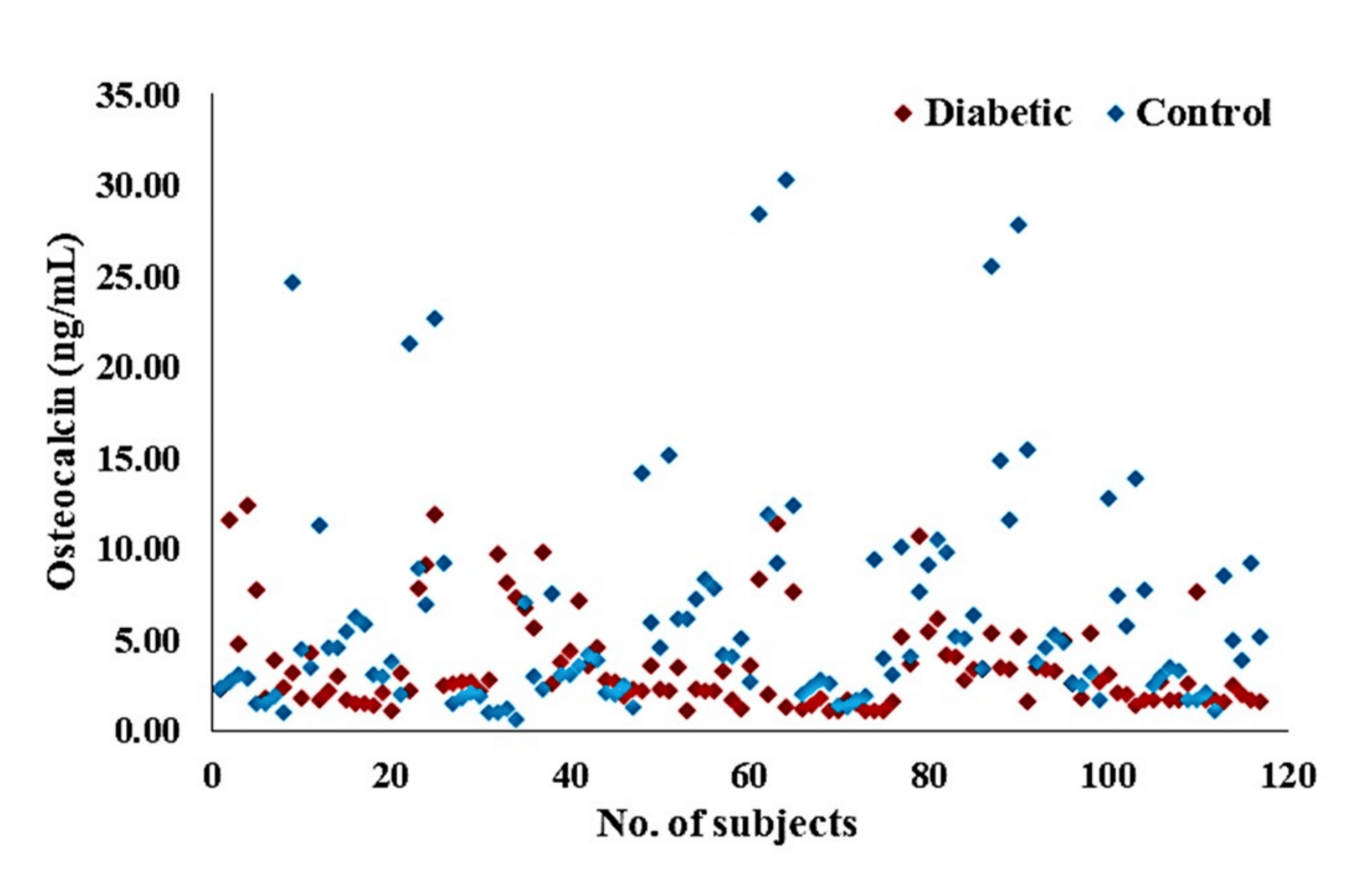
Scatter diagram showing the level of osteocalcin of the study population

Osteocalcin levels, however, were significantly (p<0.0001) lower in the diabetic group (7.07 ± 3.80) than in the control group (20.41 ± 13.50 ng/mL) (Figure 2).

Correlation of osteocalcin with glycemic parameters

**Figure 3:**
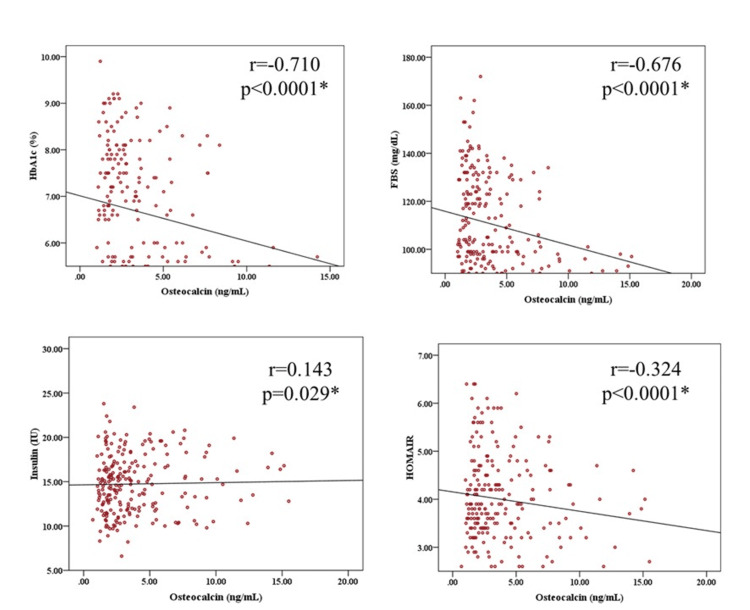
Relationship between osteocalcin and HbA1c, fasting sugar insulin, and insulin resistance The Pearson correlation coefficient was used to see the association between the two variables. HbA1c: glycated hemoglobin, FBS: fasting blood sugar, HOMA-IR: Homeostatic Model Assessment for Insulin Resistance

Pearson correlation analysis revealed that osteocalcin levels were inversely correlated with HbA1c (r=-0.710, p<0.0001) and FBS (r=-0.676, p < 0.0001). However, there was a positive but weak correlation between osteocalcin and insulin levels (r=0.143, p=0.029). Osteocalcin was also inversely correlated with HOMA-IR (r=-0.324, p<0.0001) (Figure 3).

## Discussion

The present study aimed to investigate the relationship between osteocalcin, a bone-derived hormone, and glycemic parameters in T2DM patients compared to healthy controls. The results demonstrated significantly lower levels of osteocalcin in diabetic patients, along with a significant association between osteocalcin and glycemic parameters such as FBS, insulin, and HbA1c. These findings support a role for osteocalcin in glucose metabolism and insulin sensitivity, thus positioning the skeleton as an endocrine organ involved in metabolic regulation.

The present study findings demonstrated that osteocalcin was significantly decreased (threefold) in the T2DM patients than in the control group. This is consistent with previous studies that revealed lower osteocalcin levels are associated with impaired glucose metabolism in T2DM patients [5,14]. Osteocalcin is produced by osteoblasts and has been reported to influence insulin secretion and sensitivity through its undercarboxylated form. Studies have shown that higher osteocalcin levels are correlated with improved β-cell function and insulin sensitivity [15,16], which may help explain the inverse relationship observed between osteocalcin and glucose levels in diabetic patients.

Previous studies have demonstrated that osteocalcin levels are significantly lower in individuals with T2DM compared to non-diabetic controls [5]. This reduction in osteocalcin levels has been attributed to the detrimental effects of diabetes on bone health, particularly the impairment of osteoblast function and bone formation. Diabetes-induced reductions in osteoblast differentiation and activity result in decreased osteocalcin secretion, which may contribute to the dysregulation of glucose metabolism seen in diabetic patients [17]. Furthermore, osteocalcin levels have been found to inversely correlate with key glycemic markers, such as FBS and HbA1c, suggesting that reduced osteocalcin levels may be associated with poor glycemic control in T2DM patients [5].

The correlation analysis showed a significant negative correlation between osteocalcin and both HbA1c (r=-0.7, p<0.01) and FBS (r=-0.6, p<0.01). These findings suggest that osteocalcin may play a protective role in maintaining glucose homeostasis, and its deficiency in T2DM could contribute to poor glycemic control. The negative association between osteocalcin and FBS supports the hypothesis that osteocalcin contributes to glucose regulation, possibly by enhancing insulin sensitivity in peripheral tissues. The diabetic patients in our study exhibited significantly higher insulin resistance, as evidenced by elevated HOMA-IR scores (4.39 ± 1.95 vs. 3.62 ± 1.82, p=0.002). Insulin levels were also significantly higher in the diabetic group compared to controls (15.41 ± 7.43 IU vs. 13.10 ± 3.42 IU, p=0.002). These findings are in agreement with the growing body of evidence that links insulin resistance to T2DM pathophysiology.

Interestingly, while osteocalcin was negatively correlated with HOMA-IR (r=-0.324, p=0.000), it was very weakly correlated with insulin levels in this study (r=0.143, p=0.029). This suggests that the relationship between osteocalcin and insulin resistance may be more complex, possibly involving multiple pathways, including those related to adiponectin, another hormone linked to both osteocalcin and glucose metabolism. Some studies have also suggested that osteocalcin’s effects on insulin sensitivity may be mediated indirectly through adipokines such as leptin and adiponectin [18,19]. In T2DM, reduced osteocalcin levels may indicate a compromised endocrine function of the bones, potentially leading to insulin resistance. Osteocalcin is known to improve insulin sensitivity, and its deficiency could disrupt glucose regulation. Variations in osteocalcin levels can result from genetic factors, age, physical activity, diet, and general health. although further research is needed to fully elucidate these mechanisms.

An interesting finding in our study was the significant difference in socioeconomic status between the diabetic and control groups (p=0.014). A larger proportion of the diabetic group came from the "poor" category, while more controls belonged to the "rich" category. Previous studies have documented an association between low socioeconomic status and a higher risk of developing T2DM [20,21], which may be due to factors such as reduced access to healthcare, unhealthy diets, and lower physical activity levels. Our results suggest that socioeconomic factors may play a role in the prevalence of T2DM and should be considered when developing prevention strategies.

Although our study provides valuable insights into the relationship between osteocalcin and T2DM, there are some limitations. We measured only undercarboxylated osteocalcin in our study, and we did not differentiate between the carboxylated and undercarboxylated forms of the hormone, which may have distinct biological effects on glucose metabolism. Future research should focus on these different isoforms to gain a deeper understanding of their specific roles in metabolic health. The present findings reinforce this potential, showing that osteocalcin may be a useful clinical marker for evaluating metabolic health and assessing the risk of T2DM complications. However, the precise clinical utility of osteocalcin as a biomarker warrants further investigation through large-scale, longitudinal studies.

## Conclusions

This study demonstrated that osteocalcin levels are significantly lower in T2DM patients compared to healthy controls, and these levels are inversely correlated with key glycemic parameters, including HbA1c and FBS. These findings support the growing body of evidence that osteocalcin plays an important role in glucose regulation and insulin sensitivity. Given its potential role as a biomarker for metabolic health, osteocalcin warrants further investigation as both a diagnostic tool and therapeutic target in the management of T2DM. The inverse relationship between osteocalcin and glycemic parameters means osteocalcin could serve as a potential biomarker for glucose homeostasis in T2DM patients.
